# Culture Strategy Determines the Differentiation Status of Sweat Gland Cells

**DOI:** 10.3390/cells14201643

**Published:** 2025-10-21

**Authors:** Henri De Koninck, Karel Ferland, Martin A. Barbier, Danielle Larouche, Lucie Germain

**Affiliations:** 1 The Tissue Engineering Laboratory (LOEX), Université Laval’s Research Center, Quebec, QC G1V 0A6, Canada; henri.de-koninck.1@ulaval.ca (H.D.K.); karel.ferland.1@ulaval.ca (K.F.); martin-alexandre.barbier.1@ulaval.ca (M.A.B.); danielle.larouche@crchudequebec.ulaval.ca (D.L.); 2 Department of Surgery, Faculty of Medicine, Université Laval, Quebec, QC G1V 0A6, Canada; 3 Regenerative Medicine Division, CHU de Québec-Université Laval Research Centre, Quebec, QC G1J 1Z4, Canada

**Keywords:** sweat gland, cell culture, spheroids, cell differentiation, skin, tissue engineering

## Abstract

Reliable methods for the isolation and culture of human eccrine sweat gland cells (SGCs) are essential for studying glandular biology and developing tissue-engineered skin substitutes (TESs) that restore full skin function. However, maintaining the glandular phenotype of SGCs *in vitro* remains a major challenge. In this study, we present an optimized isolation protocol combining enzymatic digestion with mechanical separation to improve SGC yield and purity, while also enabling keratinocyte isolation from a single human skin biopsy. We then evaluated two culture strategies, 2D monolayers and 3D spheroids, to determine their impact on SGC identity and proliferation. While 2D culture supported cell expansion, SGCs and keratinocytes exhibited highly similar marker expression profiles, with the absence of functional SGC markers (AQP5, α-SMA) reflecting a shift toward less differentiated phenotypes. In contrast, SGCs cultured in 3D spheroids preserved the expression of SGC-specific markers (AQP5, K18, α-SMA), distinguishing them from keratinocytes; however, their growth and structural organization were suboptimal under these 3D conditions. Moreover, SGCs expanded in 2D did not regain their glandular features when reintroduced into 3D culture, suggesting potential limitations in phenotype recovery. These results highlight the need for improved culture systems that maintain SGC identity while supporting expansion. Advancing such methods is a critical step toward integrating functional sweat glands into TESs and achieving complete skin regeneration for clinical applications.

## 1. Introduction

Sweat glands are specialized exocrine structures embedded within the dermis, responsible for thermoregulation, skin hydration, and contributing to the innate immune defense of the skin [1,2,3,4]. In humans, two primary types exist: eccrine glands, which are distributed across most of the body and play a critical role in temperature regulation through perspiration, and apocrine glands, located mainly in the axillary and genital regions, associated with scent release [5]. Beyond their physiological functions, sweat glands are increasingly recognized for their involvement in wound healing, electrolyte balance, and skin homeostasis [6,7,8,9].

The ability to isolate and culture eccrine sweat gland cells (SGCs) holds significant scientific and clinical relevance. From a basic research perspective, cultured SGCs enable the study of gland development, differentiation, and regeneration. Clinically, they offer potential for skin tissue engineering applications, particularly for the treatment of extensive burns, where traditional skin substitutes lack skin appendages such as sweat glands [10,11]. Furthermore, SGCs are a promising cell source for regenerative medicine, disease modeling (e.g., in hyperhidrosis or anhidrosis), and drug testing platforms [12].

Several methods have been developed to isolate and culture SGCs, each with distinct advantages and limitations. The most common strategy involves enzymatic digestion, typically using collagenase alone or in combination with dispase or trypsin-EDTA, followed by manual isolation of the sweat glands using a pipette under a microscope [13,14,15,16]. This method is efficient and yields a high rate of SGC culture establishment, though it may compromise tissue sweat gland structure if not carefully controlled. Mechanical isolation is another approach, involving repeated sweat gland microdissection under stereomicroscopy [17,18]. While this method is more labor-intensive and technically demanding, it better preserves the gland’s structural integrity, which can be crucial for certain downstream applications. Lastly, some protocols use combined methods, integrating enzymatic digestion with mechanical manipulation to balance yield and structural preservation. For example, dispase II treatment followed by tissue shredding and collagenase digestion has proven effective in optimizing SGC recovery and viability [19].

Culture conditions for SGCs also vary widely across protocols, with some relying on the use of feeder layers or substrates such as Matrigel or polycarbonate filters, while others use serum-free media enriched with growth factors like epidermal growth factor (EGF), bovine pituitary extract, insulin, and hydrocortisone to promote proliferation and preserve some markers (K14, K18 and CEA) of SGC cellular phenotype [14,15,20]. More recently, three-dimensional (3D) SGC culture systems and organoid models have gained prominence for their capacity to better replicate the native microenvironment and encourage gland-like structure formation [13,21,22]. Despite these advances, maintaining long-term SGC phenotype and function *in vitro* remains challenging. Under conventional two-dimensional (2D) culture conditions, SGCs rapidly lose the expression of key functional markers (AQP5, α-SMA, CHRM3) [21,23]. While some sweat gland-associated markers may still be detectable in 2D culture, many are also expressed by keratinocytes cultured in similar conditions, making it difficult to distinguish between the two cell types. Notably, direct comparative analyses of SGC and keratinocyte marker expression in 2D culture are virtually absent from the literature. Although several studies assert that SGCs can retain their phenotype in long-term 2D culture [14,15], the lack of evidence for a specific molecular functionality signature makes their reliable identification difficult. These limitations highlight the need for culture methods that support sustained SGC proliferation while preserving their phenotype and functional characteristics over extended culture periods.

In this context, the development and refinement of techniques for SGC isolation and culture are not only pivotal for advancing our understanding of glandular biology but also critical for future translational applications in skin reconstruction and regenerative medicine. This study seeks to optimize SGC isolation and to identify the optimal culture method for enhanced SGC proliferation, all while preserving their glandular phenotype. Accordingly, the optimized protocol was designed to enable the isolation of additional cell types, specifically keratinocytes, thereby maximizing the utility of human skin samples while also being compatible with established TES production processes [24,25]. Following isolation with the optimized protocol, SGCs were cultured under 2D conditions or as 3D spheroids and subsequently characterized primarily by immunofluorescence.

## 2. Materials and Methods

### 2.1. Ethical Considerations

These experiments were conducted in agreement with the Declaration of Helsinki and our institution’s guidelines. This study was approved by our institution’s protection of human participants (Comité d’éthique de la recherche du CHU de Québec—Université Laval, No. 2012-1251 approved yearly since 2012). Written informed consent was obtained for the use of skin tissues for research and educational purposes.

### 2.2. Sweat Gland Isolation

**Figure 1:**
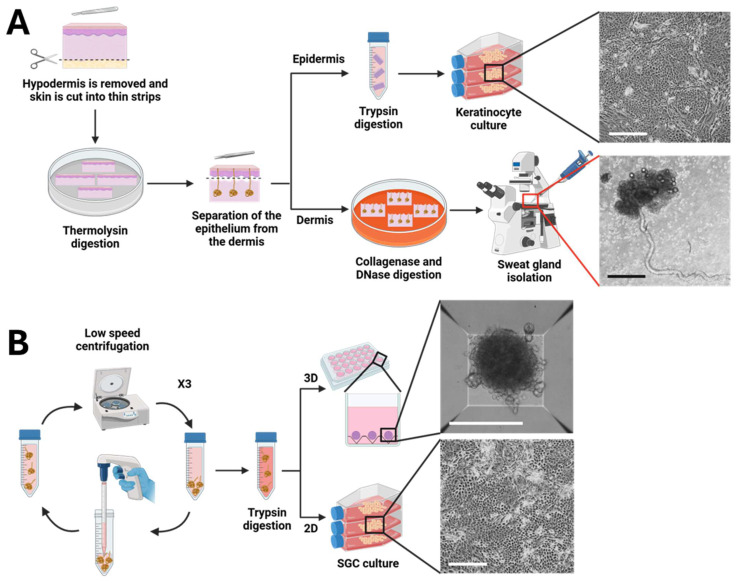
Optimized protocol for the isolation and purification of sweat gland cells. (**A**) Extraction of sweat gland cells (SGCs) from human skin samples. Phase-contrast micrographs of keratinocytes in 2D culture and an isolated sweat gland (40×, scale bar = 500 μm). (**B**) Purification and culture of SGCs. Phase-contrast micrographs of SGCs cultured in 2D (40×, scale bar = 500 μm) and 3D spheroid culture (100×, scale bar = 300 μm). Created in BioRender. De Koninck, H. (2025) https://BioRender.com/jquv4w3 (accessed on 27 August 2025).

The SGC isolation and purification protocol is summarized in Figure 1. Skin biopsies were obtained from healthy donors undergoing surgical resection, primarily lipectomies in women between the ages of 30 and 65 years, following informed consent. Adipose tissue was removed using scissors, and the remaining tissue was washed ten times for 2 min each with phosphate-buffered saline (PBS) (Thermo Fisher Scientific, Waltham, MA, USA). The tissue was then cut into 3 mm strips and incubated for 4 h at 37 °C in a solution of 500 µg/mL thermolysin (Thermo Fisher Scientific) dissolved in HEPES buffer (0.01 M 4-(2-hydroxyethyl)-1-piperazineethanesulfonic acid (HEPES; MP Biomedicals, Santa Ana, CA, USA) solutions with 0.67 mM KCl (Thermo Fisher Scientific), 0.14 M NaCl (Thermo Fisher Scientific) and 1 mM CaCl_2_ (Sigma Aldrich, Saint-Louis, MO, USA). The epidermis and the hair follicles were then carefully separated from the dermis using forceps.

Epithelial cells were isolated by digesting the epithelium in Trypsin (0.05%) (Gibco, Grand Island, NY, USA—EDTA (0.01%) (Sigma, Oakville, ON, Canada) solution at 37 °C for 25 min under constant agitation then cultured. To isolate SGCs, the dermis was minced into 1 mm × 1 mm cubes with a scalpel and incubated for 16 h at 37 °C on a rocking platform in Dulbecco-Vogt modified Eagle medium (DMEM; Gibco, Waltham, MA, USA) supplemented with 2 U/mL collagenase H (Roche, Mississauga, ON, Canada) and 25 µg/mL DNase (Roche). Under a low-magnification phase contrast microscope, intact sweat glands were manually harvested using 200 µL pipet tips with wide openings and placed into keratinocyte culture medium (Dulbecco-Vogt modified Eagle medium (Gibco): Ham’s F12 (Gibco), ratio 3:1, supplemented with 24.25 μg/mL adenine (Sigma-Aldrich), 5 μg/mL insulin (Sigma-Aldrich), 0.4 μg/mL hydrocortisone (Galenova, Saint-Hyacinthe, QC, Canada), 0.212 μg/mL isoproterenol hydrochloride (Sigma-Aldrich), 5% bovine HyClone FetalClone II serum (GE Healthcare, Chicago, IL, USA), 10 ng/mL human epidermal growth factor (Austral Biologicals, San Ramon, CA, USA), 100 U/mL penicillin (Sigma-Aldrich) and 25 μg/mL gentamicin (Gemini Bio, West Sacramento, CA, USA). To eliminate dermal fibroblast contamination, sweat glands were washed three times in keratinocyte culture medium using low-force centrifugation (10× *g* for 1 min). The isolated sweat glands were then digested in a trypsin (0.05%)—EDTA (0.01%) solution for 25 min at 37 °C with constant agitation. Debris was removed using a 100 µm filter to obtain purified primary SGCs.

### 2.3. Cell Culture

Primary cells (keratinocytes and SGCs) were either used directly for experiments or cryopreserved for later use. For cryopreservation, cells were resuspended in a freezing solution containing 90% Avantador Seradigm FB Essence serum (Avantor, Radnor, PA, USA) and 10% dimethyl sulfoxide (DMSO) (Sigma-Aldrich). Vials were placed in a Mr. Frosty freezing container (Thermo Fisher Scientific, Waltham, MA, USA) and incubated at −80 °C overnight. The vials were then transferred to a liquid nitrogen storage tank.

For thawing, frozen cells were rapidly warmed in a 37 °C water bath. The cell suspension was diluted 1:10 by adding nine volumes of culture medium, and the solution was centrifuged at 300× *g* for 10 min to pellet the cells and remove the cryopreservation medium.

Freshly isolated or thawed keratinocytes and SGCs were cultured in monolayer (2D) on plastic culture dishes containing a feeder layer composed of irradiated human fibroblasts, in keratinocyte culture medium. Between each step, cells were counted using a Coulter cell counter (Beckman Coulter, Mississauga, ON, Canada) and viability was assessed using trypan blue staining. Cultured cells were detached from the culture dish through incubation for 15 min at 37 °C in a trypsin (0.05%)—EDTA (0.01%) solution. Cell proliferation in 2D cultures was assessed by the population doubling time, calculated using the following formula:

(1) $$PDoub=\frac{T\cdot \ln2}{\ln\frac{C_{f}}{C_{i}}}$$

Here, *PDoub* denotes the population doubling time, *T* corresponds to the culture duration in days, *Ci* represents the initial number of seeded cells, and *Cf* indicates the final number of cells quantified at the end of the culture period.

### 2.4. Spheroid Formation

AggreWell plates (AggreWell 800 24-well plate, StemCell Technologies, Vancouver, BC, Canada) were prepared according to the manufacturer’s recommendations. Briefly, each well was pre-treated by adding 500 µL of anti-adherence rinsing solution, followed by centrifugation at 1300× *g* for 5 min to remove any trapped air bubbles. The rinsing solution was then aspirated, and the wells were washed with 2 mL of keratinocyte culture medium. Sweat gland cells were seeded at a density of 500 to 1000 cells per microwell. After seeding, the plate was immediately centrifuged at 100× *g* for 3 min. Every 24 h, half of the culture medium was replaced with fresh medium and spheroid formation was monitored through visualization under a microscope.

### 2.5. Immunofluorescence

Harvested spheroids were embedded for cryosectioning by gently resuspending them in Tissue-Tek optimal cutting temperature compound (OCT; Sakura, Finetek, Torrance, CA, USA) and flash-freezing the mixture in liquid nitrogen. Skin tissue samples were prepared similarly. Six µm-thick sections were cut from the frozen blocks using a cryostat (Leica, Wetzlar, Germany).

Prior to immunolabelling, a heat-induced antigen retrieval step was performed by incubating the sections at 37 °C for 30 min. The sections were subsequently fixed and permeabilized using cold acetone at a temperature of −20 °C for 10 min. For immunolabelling, sections were first incubated in a blocking solution (PBS supplemented with 2% *w*/*v* bovine serum albumin) for 30 min. Primary and/or secondary antibodies were diluted to their working concentration in blocking buffer and applied to the sections for a 1 h incubation at room temperature (Supplementary Table S1). Nuclei were counterstained using Hoechst (Sigma-Aldrich, Saint-Louis, MO, USA). A series of three 2 min washes in PBS was performed following each incubation step.

## 3. Results

### 3.1. Protocol Optimization

**Figure 2:**
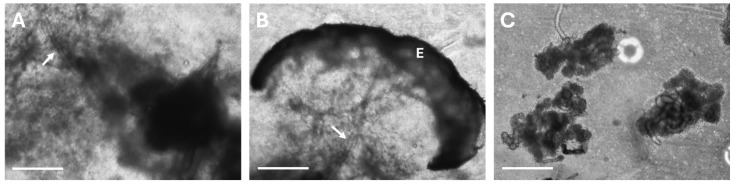
Impact of enzymatic digestion conditions and epithelium removal on tissue integrity during sweat gland isolation. Human skin samples digested with low-concentration collagenase (**A**) and without epithelium removal (**B**) are compared to dermal samples treated with a combined high-concentration collagenase and DNase digestion following epithelium removal (**C**). White arrows indicate sweat gland ducts and E indicates the epithelium. Phase-contrast micrographs (40×, scale bar = 500 μm).

The sweat gland isolation protocol was optimized to increase efficiency, improve cell recovery, and expand the range of cell types retrieved from human skin biopsies. In the initial procedure, skin was minced and digested in low-concentration collagenase, and glands were manually extracted from the remaining dermis. First, the collagenase concentration was increased, which enhanced dermal degradation and facilitated the release of intact glands (Figure 2A,C, white arrows). Second, DNase was added to the digestion mixture, reducing viscosity by degrading extracellular DNA and preventing gland adhesion to other skin components, thereby improving recovery efficiency and minimizing contaminations by other cell types. Finally, an epithelium removal step was introduced prior to dermal digestion, using thermolysin treatment followed by mechanical peeling. This modification not only improved dermal digestion and reduced contamination with keratinocytes in the SGC population (Figure 2B,C), but also enabled the parallel isolation and culture of keratinocytes. These keratinocytes were used for direct comparison with SGCs. Together, these changes resulted in a more versatile and reproducible protocol for the isolation of sweat glands and associated skin cell populations.

### 3.2. SGC Immunolabeling and 2D Culture Characterization

**Figure 3:**
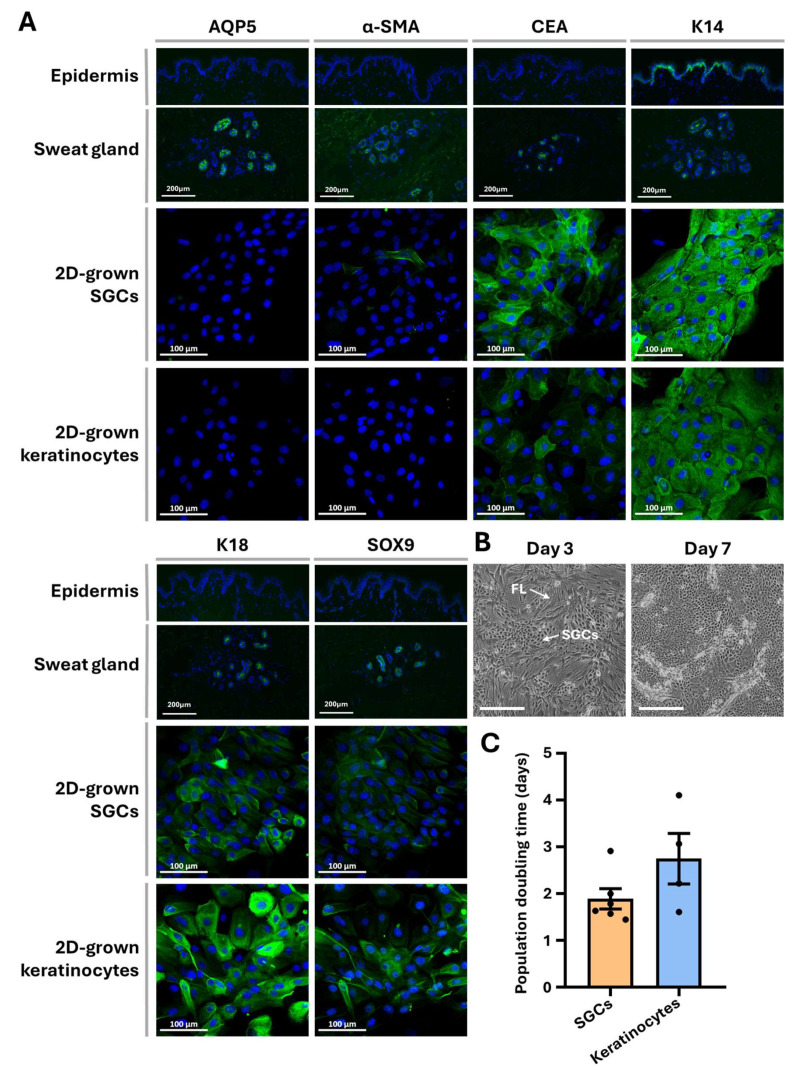
Proliferation and marker expression of SGCs in human skin and 2D primary culture. (**A**) Immunofluorescence staining for SGC markers (green), namely aquaporin 5 (AQP5), α-smooth muscle actin (α-SMA), carcinoembryonic antigen (CEA), keratin 18 (K18), SOX9, and keratin 14 (K14), in skin sections compared with 2D cultured SGCs and keratinocytes. Cell nuclei were counterstained with Hoechst (blue). (**B**) Phase-contrast micrographs of SGCs at days 3 and 7 of culture. FL indicates the fibroblast feeder layer and SGCs indicates the cells (40×, scale bar = 500 μm). (**C**) Population doubling time of SGCs and keratinocytes in primary 2D culture (SGCs: number of cell donors (N) = 6, keratinocytes: N = 4, *p*-value > 0.1 in Mann–Whitney test).

SGCs obtained through the established isolation protocol and maintained under 2D culture conditions were characterized using immunofluorescence staining. The expression patterns of six sweat gland-associated markers were compared on normal human skin (NHS) tissue section (epidermis and sweat glands), 2D-cultured keratinocytes, and 2D-cultured SGCs (Figure 3A). Aquaporin 5 (AQP5), a water channel protein, and keratin 18 (K18) are typically expressed in both dark and clear cells of the sweat gland secretory region [8]. α-smooth muscle actin (α-SMA) serves as a marker for myoepithelial cells in the secretory portion, while carcinoembryonic antigen (CEA) is expressed by secretory cells and the duct cells alike [26,27,28]. Keratin 14 (K14) localizes to basal cells of the sweat duct, as well as the myoepithelial cells, and SOX9 is a recognized marker of epithelial stem/progenitor cells [29,30].

In NHS tissue, all six markers were detected within sweat glands, whereas the NHS epidermis expressed only K14 in the basal layer. Following 2D culture, SGCs exhibited complete loss of AQP5 expression and displayed α-SMA positivity only in a minority of cells. In contrast, the expression of CEA, K18, SOX9, and K14 remained largely unchanged. Keratinocytes cultured under identical 2D conditions demonstrated a similar marker expression profile compared with 2D-cultured SGCs, although CEA expression was comparatively lower in keratinocytes. These results indicate that both SGCs and keratinocytes undergo phenotypic modifications under 2D culture conditions, with cultured SGCs acquiring a phenotype more closely resembling that of cultured keratinocytes.

SGC proliferative capacity was further assessed microscopically (Figure 3B) and by determining population doubling time during primary culture (Figure 3C). Comparative analysis revealed no statistically significant difference in proliferation rate between SGCs and keratinocytes (*p*-value > 0.1), indicating that 2D culture conditions are compatible with sustaining SGC proliferation.

### 3.3. 3D Culture Characterization

**Figure 4:**
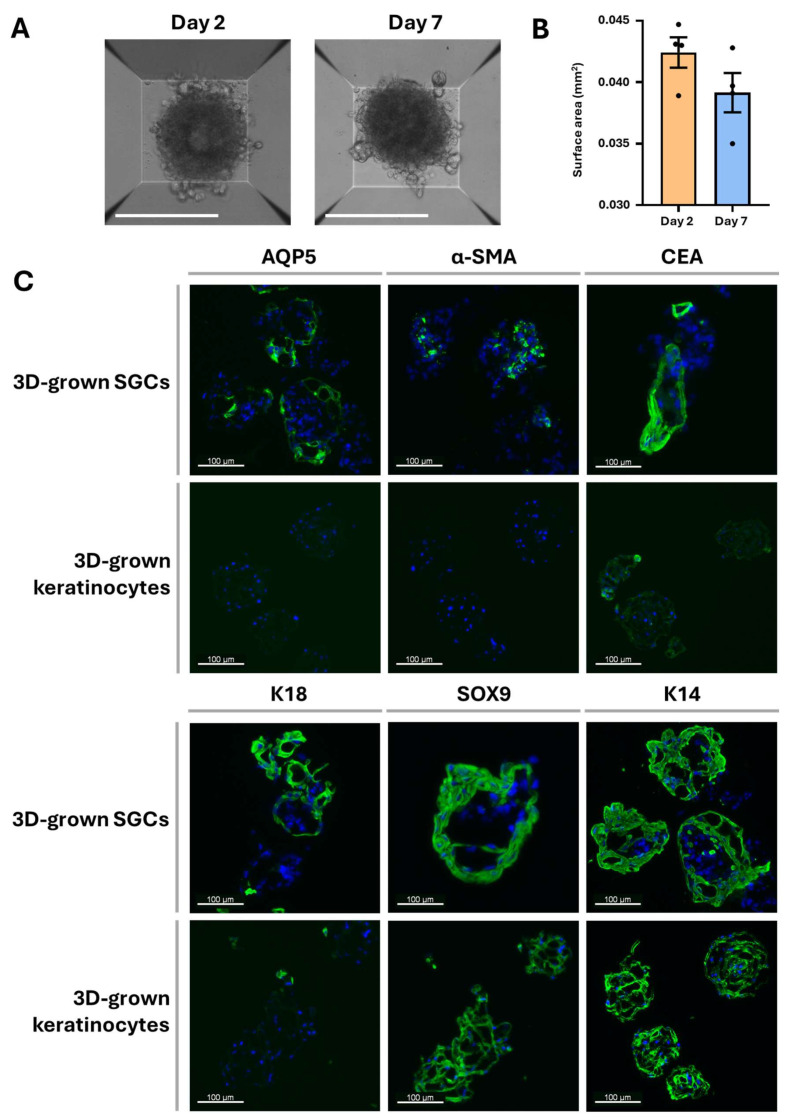
Spheroid growth and marker expression of SGCs in 3D culture of freshly isolated cells. (**A**) Phase-contrast micrographs of SGC spheroids at days 2 and 7 of culture (100×, scale bar = 300 μm). (**B**) Size comparison of spheroids over time (n = 4). (**C**) Immunofluorescence staining for SGC markers (green) in spheroids generated from SGCs and keratinocytes. Cell nuclei were counterstained with Hoechst (blue).

To evaluate the impact of 3D culture conditions on the differentiation of SGCs, SGC spheroids were analyzed using phase-contrast microscopy and immunofluorescence. After 7 days in 3D culture, no change in SGC spheroid size was observed over time, suggesting that the cells within the spheroids exhibited minimal to no proliferation (Figure 4A,B). Despite this limited growth, 3D-cultured SGCs expressed all tested sweat gland markers, including AQP5, α-SMA, CEA, K18, SOX9, and K14 (Figure 4C).

The spheroids exhibited a disorganized architecture, with no detectable basement membrane protein expression after one week in culture (Figure S1). In contrast, keratinocytes cultured in 3D expressed SOX9 and K14 at levels similar to those in 3D-cultured SGCs, but exhibited lower expression of CEA and K18, and lacked expression of AQP5 and α-SMA. Altogether, these findings suggest that the 3D culture environment influences SGC phenotype, promoting the maintenance of a marker expression profile more closely resembling that of native NHS sweat glands, compared to 2D-cultured SGCs, where many SGC markers were lost. However, unlike in SGCs, the keratinocyte marker expression profile remained unchanged between 2D and 3D keratinocyte cultures.

### 3.4. 3D Culture Phenotype Recovery

**Figure 5:**
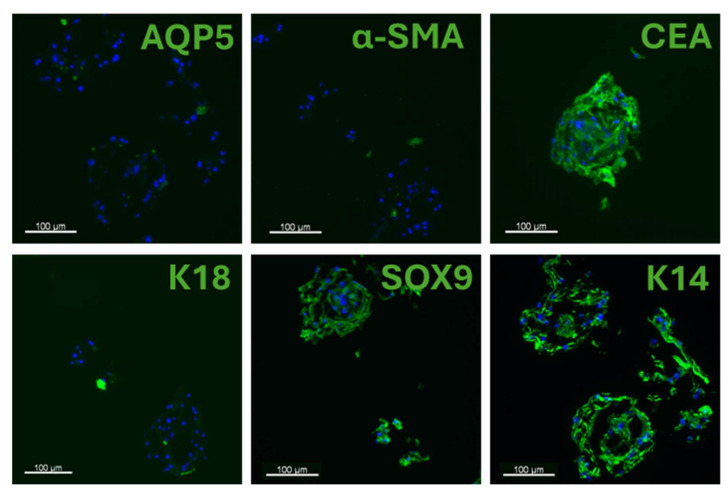
Effect of 3D culture on marker expression in SGCs previously grown in monolayer (2D). Immunofluorescence staining for SGC markers (green) in spheroids generated from SGCs cultured for one passage in 2D prior to 3D spheroid formation. Cell nuclei were counterstained with Hoechst (blue).

To assess whether 2D-cultured SGCs could reacquire the phenotypic characteristics of differentiated SGCs, cells were first cultured in 2D for one passage and subsequently transferred to 3D culture conditions. The resulting spheroids were analyzed via immunofluorescence. SGCs transitioned from 2D to 3D cultures expressed CEA, SOX9, and K14, but lacked expression of AQP5 and α-SMA (Figure 5). This marker expression profile closely resembled that of 2D primary culture of SGCs, suggesting that introducing 3D culture following prior 2D expansion is insufficient to restore the markers observed in native glands. This differential expression indicates that while 2D culture induces a partial phenotypic shift toward a keratinocyte-like state, the resulting phenotype is not entirely equivalent to that of keratinocytes.

## 4. Discussion

A major challenge in the engineering of fully functional human skin lies in the regeneration of skin appendages, which fulfill various essential roles in skin physiology. Among these, sweat glands are particularly important for body’s thermoregulation. However, the mechanisms underlying the development of these skin appendages remain incompletely understood, underscoring the need for improved *in vitro* models to facilitate their analysis. In this study, we present an optimized protocol for the isolation of SGCs that also allows for the parallel recovery of keratinocytes from the same human skin biopsies. The method combines sequential enzymatic digestions, beginning with thermolysin, for epithelium removal from the dermis, followed by incubation of the dermis in a high-concentration solution of collagenase and DNase, before manual recovery of the sweat glands under a phase-contrast microscope and gland purification through low-speed centrifugations (Figure 1 and Figure 2). These refinements enhanced dermal breakdown, reduced digestion mixture viscosity, and lowered keratinocyte contamination in the SGC fraction. Importantly, separating the epithelium enabled the simultaneous isolation and culture of keratinocytes from the epithelial fraction (epidermis and hair follicles) and the SGCs from the dermal fraction. This facilitated direct comparisons with SGCs from the same donor, improving the utilization of skin biopsies, and providing a valuable cell source for tissue-engineered skin (TES) production.

We further evaluated the influence of two culture conditions on SGC proliferation and differentiation by assessing the expression of sweat gland-associated markers via immunofluorescence. A key aspect of our approach involved a direct comparison between the expression profiles of SGCs and keratinocytes, cultured in parallel under identical conditions to minimize variables associated with inter-donor variability. A feeder layer was used to extend epithelial cell lifespan and maintain stem cell potential during culture [31,32]. This represents, to our knowledge, the most comprehensive analysis of this kind to date. Our findings indicate that under 2D culture conditions, SGCs exhibit a tendency to lose their specific markers and adopt keratinocyte-like characteristics, whereas 3D culture in spheroids promotes the maintenance of a glandular phenotype. However, rapid SGC expansion was limited in the 3D conditions tested.

In our study, cultured SGCs and keratinocytes exhibited highly similar marker expression profiles, particularly under monolayer (2D) culture conditions, likely due to their shared epithelial origin (Figure 3A). This phenotypic overlap may be attributed to the inherent plasticity of SGCs, which can differentiate into keratinocytes during epidermal regeneration following injury [6,7,9]. We observed that when cultured in conditions that favors lower differentiation states (2D), SGCs tend to downregulate markers typically associated with sweat gland identity and functionality, such as AQP5 and α-SMA. Interestingly, under the same conditions, keratinocytes expressing markers generally associated with sweat glands are observed, such as CEA, SOX9, and K18 (Figure 3A), which are not phenotypes of differentiated keratinocytes. The enrichment of less differentiated cells because they proliferate faster may explain this phenotypic shift observed for both cell types (Figure 3B,C). The loss of AQP5 and α-SMA in SGCs is consistent with this hypothesis, as both proteins are functionally involved in sweat secretion, a function only present in differentiated SGCs [1,33]. Likewise, the ectopic expression of CEA and K18, which are markers found during fetal skin development but absent in adult epidermis, supports the idea of a dominant proliferative cell population overtaking cultures and exhibiting a less differentiated phenotype [29,34,35]. The upregulation of SOX9 is particularly noteworthy, as several studies have demonstrated that SOX9 promotes proliferation and suppresses differentiation in epidermal keratinocytes and hair follicle cells [36,37]. It plays a key role in establishing the stem cell niche and maintaining cellular stemness [30,38]. Moreover, its cytoplasmic localization has been associated with increased proliferation and a less differentiated state in epithelial cells [39,40]. Thus, its expression in both SGCs and keratinocytes under 2D conditions reinforces the notion of a significant dilution of differentiated cells in a proliferative population. Collectively, these findings suggest that 2D culture promotes a reprogramming in SGCs cultures, rendering them phenotypically similar to cultured keratinocytes. However, this resemblance in marker expression prevents reliable identification of SGCs within the cultures, thereby raising uncertainty about cell identity and culture purity. As a result, it became necessary to establish an alternative culture strategy to more accurately distinguish and characterize SGCs.

To evaluate SGC behavior in a context that favors 3D structural organization, we employed a spheroid-based 3D culture system. When generated from freshly isolated SGCs, spheroids expressed AQP5, K18, and α-SMA, which are markers found in native sweat gland, enabling clear distinction with keratinocyte spheroids, which showed minimal or no expression of these markers (Figure 4C). This approach allowed us to confirm the identity of the isolated cells and suggested that the commitment to the differentiation pathway toward the sweat gland lineage was maintained throughout the culture period. However, no evidence of structural remodeling or spatial reorganization among the different SGC subtypes was observed. The spheroids exhibited a disorganized architecture, and the basement membrane was absent after one week in culture (Figure S1). Furthermore, minimal cell growth was detected, indicating that while differentiated SGCs aggregate and remain within the spheroids, only a small subpopulation—presumably stem-like cells— may undergo limited proliferation (Figure 4A,B). Thus, our results suggests that although this 3D culture system provides a reliable platform for SGC identification and short-term phenotype maintenance, it lacks the capacity to support substantial cell expansion.

To assess the regenerative capacity of SGCs, we first expanded the cells for one passage in 2D monolayer culture, then subsequently used them to generate spheroids. After one week in 3D culture, the spheroids failed to express key markers characteristic of differentiated SGCs (AQP5, α-SMA; Figure 5)—markers that were expressed in SGC spheroids in primary culture. This absence of marker expression, along with the lack of evident structural remodeling within the spheroids, suggests that the phenotypic change induced in SGCs by 2D culture is difficult to reverse, thereby compromising their capacity to differentiate into SGCs upon reintroduction into a 3D environment. Previous studies have shown that more complex 3D culture systems, including hydrogels and organoid models, enhance the formation of gland-like structures [13,21,41]. Additionally, signals present after *in vivo* grafting also appear to be key factors in the formation of *bona fide* sweat glands [20,22,42]. In any case, our study highlights that the primary culture conditions represent a critical step for the successful regeneration of functional sweat glands by tissue engineering.

## 5. Conclusions

This study establishes an optimized protocol for sweat gland cell isolation that also permits the simultaneous recovery of keratinocytes, enhancing sample use, reducing cross-culture contamination, and supporting applications in comparative studies and eventually in tissue-engineered skin production. Our findings highlight the challenges of maintaining the glandular phenotype of SGCs *in vitro*. While 2D culture promotes proliferation of stem-like cells, diluting differentiated SGCs and thus displaying phenotypic overlap with keratinocytes, 3D spheroid culture preserves SGC identity but fails to support long-term growth or structural organization. These findings underscore the need for more advanced culture systems to support functional sweat gland regeneration. Ultimately, integrating SGCs into TESs and grafting them *in vivo* may provide the necessary cues to restore sweat gland function.

## Data Availability

The data presented in this study are available upon request to the corresponding author.

## Supplementary Materials

The following supporting information can be downloaded at https://www.mdpi.com/article/10.3390/cells14201643/s1, Figure S1: Basement membrane characterization in sweat glands and 3D-cultured SGCs. Immunofluorescence staining for established basement membrane markers (green), namely laminin subunit γ-1 (LAMC1), integrin α-6 (ITGA6), and collagen type VII (COL7A1), in sweat glands in skin sections and in spheroids generated from SGCs. Cell nuclei were counterstained with Hoechst (blue); Table S1: List of antibodies.

## Abbreviations

The following abbreviations are used in this manuscript:

SGC	Sweat gland cell
TES	Tissue-engineered skin substitute
EGF	Epidermal growth factor
NHS	Normal human skin
AQP5	Aquaporin 5
α-SMA	α-smooth muscle actin
CEA	Carcinoembryonic antigen
K14	Keratin 14
K18	Keratin 18
