# Supplementary material for: Culture Strategy Determines the Differentiation Status of Sweat Gland Cells
Source: Cells. 2025 Oct 21;14(20):1643. doi: 10.3390/cells14201643 (PMC12563307; doi:10.3390/cells14201643)
Supplement: Supplementary file 1 [file cells-14-01643-s001.zip › cells-3872423-supplementary.pdf]

Supplementary Material:

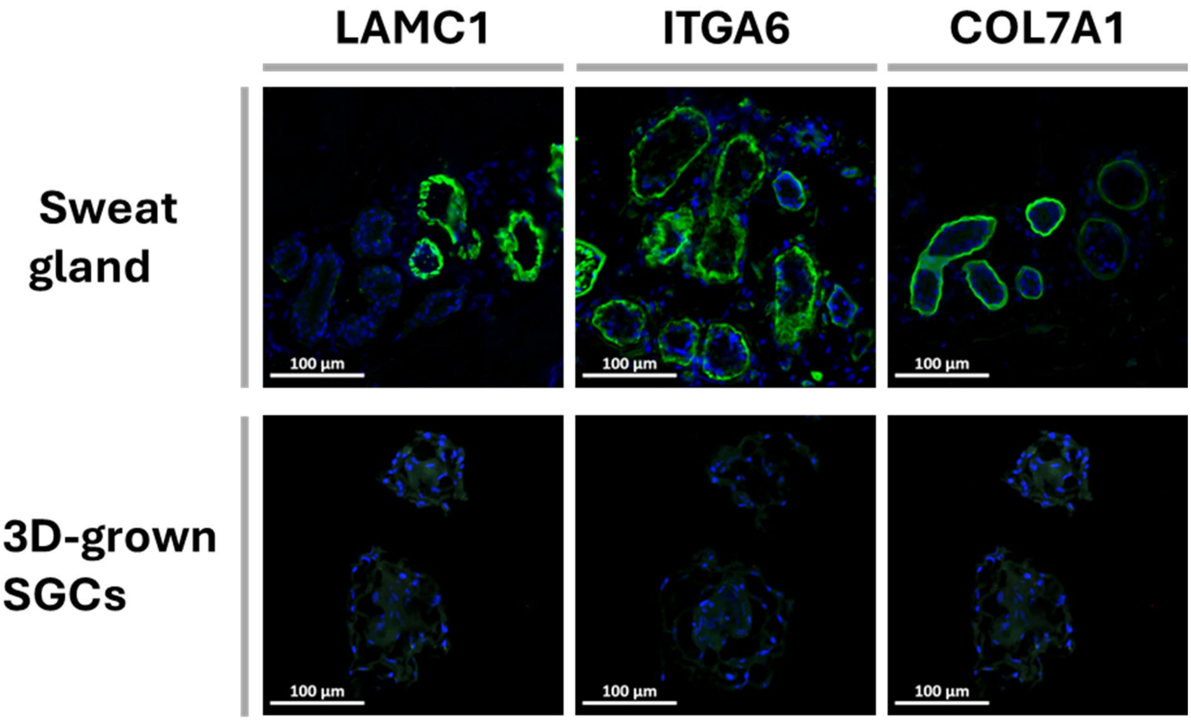

Figure S1. Basement membrane characterization in sweat glands and 3D-cultured SGCs. Immunofluorescence staining for established basement membrane markers (green), namely laminin subunit γ-1 (LAMC1), integrin α-6 (ITGA6), and collagen type VII (COL7A1), in sweat glands in skin sections and in spheroids generated from SGCs. Cell nuclei were counterstained with Hoechst (blue).

Table S1. List of antibodies

Antigen	Origin	Isotype	Conjugate	Supplier	Catalog Number	Dilution
AQP5	Mouse	IgG1, κ	-	Santa Cruz	sc-514022	1/500
SOX9	Rabbit	IgG	-	Sigma	HPA001758	1/125
CEA	Mouse	IgG1	-	ThermoFisher	MA5-14675	1/200
α-SMA	Mouse	IgG2a, κ	-	Dako	M0851	1/400
K14	Rabbit	IgG	-	Cedarlane	CLPRB-155p	1/800
K18	Mouse	IgG1	-	ARP	03-61009	1/100
Col7A1	Rabbit	IgG	-	Calbiochem	234192	1/400
ITGA6	Mouse	IgG1	-	Serotec	MCA1457	1/100
LAMC1	Mouse	IgG1	-	Santa Cruz	sc-17751	1/500
Mouse IgG (H+L)	Donkey	IgG	AF488	Abcam	ab150101	1/1000
Rabbit IgG (H+L)	Goat	IgG	AF594	Life	A11012	1/1000
